# Identification of cancer risk assessment signature in patients with chronic obstructive pulmonary disease and exploration of the potential key genes

**DOI:** 10.1080/07853890.2022.2112070

**Published:** 2022-08-20

**Authors:** Qingzhou Guan, Peng Zhao, Yange Tian, Liping Yang, Zhenzhen Zhang, Jiansheng Li

**Affiliations:** a Academy of Chinese Medical Sciences, Henan University of Chinese Medicine, Zhengzhou, China; b Henan Key Laboratory of Chinese Medicine for Respiratory Disease, Co-Construction Collaborative Innovation Center for Chinese Medicine and Respiratory Diseases by Henan & Education Ministry of P.R. China, Henan University of Chinese Medicine, Zhengzhou, China; c School of Basic Medicine, Henan University of Chinese Medicine, Zhengzhou, China; d The First Affiliated Hospital, Henan University of Chinese Medicine, Zhengzhou, China

**Keywords:** Chronic obstructive pulmonary disease, lung cancer, qualitative transcriptional characteristics, incidence-risk score, traditional Chinese medicines

## Abstract

It is essential to assess the cancer risk for patients with chronic obstructive pulmonary disease (COPD). Comparing gene expression data from patients with lung cancer (a total of 506 samples) and those with cancer-adjacent normal lung tissues (a total of 370 samples), we generated a qualitative transcriptional signature consisting of 2046 gene pairs. The signature was verified in an evaluation dataset comprising 18 subjects with severe disease and 52 subjects with moderate disease (Wilcoxon rank-sum test; *p* = 7.33 × 10^−5^). Similar results were obtained in other independent datasets. Among the gene pairs in the signature, 326 COPD stage-related gene pairs were identified based on Spearman’s rank correlation tests and those gene pairs comprised 368 unique genes. Of these 368 genes, 16 genes were significantly dysregulated in COPD rat model data compared with control data. Some of these genes (*Dhx16*, *Upf2*, *Notch3*, *Sec61a1*, *Dyrk2*, and *Hmmr*) were altered when the COPD rat model was treated with traditional Chinese medicines (TCM), including Bufei Yishen formula, Bufei Jianpi formula, and Yiqi Zishen formula. Overall, the signature could predict the cancer incidence-risk of COPD and the identified key genes might provide guidance regarding both the treatment of COPD using TCM and the prevention of cancer in patients with COPD.

**KEY MESSAGES**
- A cancer risk assessment signature was identified in patients with COPD.
- The signature is insensitive to batch effects and is well verified.
- COPD key genes identified in this study might play a crucial role in TCM treatment and cancer prevention.

## 1. Introduction

Chronic obstructive pulmonary disease (COPD) is a common respiratory disease, characterized by airflow limitation and is incompletely reversible [1,2]. Patients with COPD suffer decline in lung function resulting in a severe compromise in the quality of life and imposing heavy economic burdens on patients, families, and society [3,4]. The overall incidence of COPD was reported to be 8.6% in China, and was as high as 13.7% for individuals aged 40 years or older [5]. In 2019, the global prevalence of COPD among people aged 30–79 years was 10.3% (95% CI 8.2–12.8) using the GOLD case definition, which translates to 391.9 million people (95% CI 312.6–487.9) [6]. Moreover, COPD is an independent high-risk factor for the occurrence of lung cancer [7,8]. Lung cancer could develop from COPD through a continuous, multi-step process whereby normal lungs advance to moderate and then severe COPD, and eventually develop into cancer [9]. However, to the best of our knowledge, there is currently no molecular signature to accurately assess the risk of cancer incidence among patients with COPD. Thus, there is significant clinical value in developing a molecular signature for assessing the incidence of COPD converting to lung cancer. Traditional Chinese medicines (TCM) have unique merits, exhibiting high efficacy and fewer adverse reactions, and some TCM have been successfully applied for the treatment of COPD in clinical settings [10–12]. For example, clinical studies have revealed Tiaobu Feishen formulae (TBFS), including Bufei Yishen formula (BYF), Bufei Jianpi formula (BJF), and Yiqi Zishen formula (YZF), had desirable pharmacological effects on COPD, such as alleviating the clinical symptoms of patients with stable COPD, reducing the exacerbation frequency, delaying acute exacerbation, and improving pulmonary function and exercise capacity [13]. Moreover, these three formulae have demonstrated beneficial effects in COPD rat model, inhibiting expression of inflammatory cytokines, protease–antiprotease imbalance, and collagen deposition [10,14–17]. Among the genes constituting the cancer risk signature, it will also be of significance to identify potential key genes that are reversed when the COPD rat model is treated with those TCMs, which might guide COPD treatment using TCM and aid in the prevention of lung cancer occurrence from COPD.

High-throughput gene detection technology has become widely applied, and various quantitative transcriptional signatures have been used in subtyping diseases and early diagnosis [8,18–21]. Nevertheless, due to batch effects, these types of signatures are not suitable for the analysis of individuals and are therefore difficult to apply them in clinical practice. Several disease signatures based on quantitative transcriptional feature, such as AlloMap^®^ [22], have already been approved by the US Food and Drug Administration (FDA). However, because of batch effects, those samples must be measured in specific laboratories, which also limits their clinical application. Qualitative transcriptional characteristics, also called within individual sample relative expression orderings (REOs) of genes, are robust solutions to the batch effect problem and suitable for individualized analysis in clinical practice [22,23]. Using the robust performance of qualitative transcriptional characteristics, researchers can merge data detected by the same or similar platforms from multiple sources to train classifier models or signatures, which would easily obtain robust signatures [2,24,25]. Furthermore, the technique is suitable for samples detected using different platforms.

Based on the unique merits of qualitative transcriptional characteristics, this study identified a cancer incidence-risk signature for patients with COPD without cancer, and the performance of the signature was verified in multiple independent datasets. Furthermore, among the genes constituting the cancer risk signature, COPD key genes that could be regulated by TCM were identified, and the value of these COPD key genes in drug treatment and cancer prevention warrants further exploration.

## 2. Materials and methods

### 2.1. Public data and preprocessing

Gene expression profiles of lung cancer and normal lung tissue samples from multiple sources were downloaded from the GEO database (Table 1). For data detected by the Affymetrix platform, the raw mRNA expression data (.CEL files) was downloaded and the Robust Multi‐array Average (RMA) algorithm was applied for preprocessing. For data detected by Illumina or Agilent platforms, the processed data were directly downloaded. All cancer samples were from surgical resection in patients with non-small cell lung carcinoma (NSCLC), while the normal samples were obtained from adjacent normal tissues of patients with lung cancer.

**Table 1.** Data analysed in this study.

GEO No.	Gene^a^	Platform	Normal sample size	Cancer sample size
GSE19804	20486	Affymetrix GPL570	60	60
GSE18842	20486	Affymetrix GPL570	45	46
GSE27262	20486	Affymetrix GPL570	25	25
GSE31210	20486	Affymetrix GPL570	20	226
GSE19188	20486	Affymetrix GPL570	65	91
GSE32863	25186	Illumina GPL6884	58	58
GSE31267	24384	Illumina GPL6947	24	–
GSE15197	18615	Agilent GPL6480	13	–
GSE40588	19595	Agilent GPL6480	60	–

^a^The number of genes detected in the corresponding dataset.

–: there is no sample in the corresponding category.

For the downloaded data, when multiple probes mapped to an identical gene, the measurement of the gene was calculated as the arithmetic mean value of the multiple probe values. When a probe mapped to none or more than one gene, the probe data were discarded.

### 2.2. COPD rat data and drug treatment

The rat data analysed in this study were obtained from our previous study [10–12,26] and a COPD model generated using Sprague–Dawley rats was prepared as previously described [27]. Briefly, the rats were exposed to cigarette smoke and repeated *Klebsiella pneumoniae* infections. In the ninth week, COPD model rats were randomly divided into five groups as shown in Supplementary Table S1. The groups of rats were intragastrically treated with normal saline (model group, 2 mL/animal), aminophylline (APL, 2.3 mg/kg), BYF, BJF, or YZF each day from weeks 9 to 20, respectively; the drug concentrations and dose of the three TCMs are shown in Supplementary Table S2. Dosages of the TCM formulae were calculated according to the clinically used dosages of adult patients and the body surface area conversion equation between human and rat: *D*_rat_=*D*_human_×(*I*_rat_/*I*_human_)×(*W*_rat_/*W*_human_)^2/3^, where *D* is dose, *I* is body shape index, and *W* is body weight. The control group rats were fed with normal saline intragastrically (2 mL). Each group included six replicates and the rats in each group were separately given the corresponding drug or normal saline treatment. All animals were handled humanely during the process of the experiment and were anaesthetized and sacrificed to obtain lung tissues on week 32. The components of BYF, BJF, YZF, and APL were described in previous studies [10–12]. Mass spectrometry and high-performance liquid chromatography fingerprint were respectively performed in previous studies to identify the main chemical constituents of BYF and BJF [28,29].

Briefly, BYF (patent: ZL.201110117578.1) is composed of 12 Chinese medicinal herbs, including *Panax ginseng C.A.Mey.* 9 g, *Astragalus mongholicus Bunge* 15 g, *Cornus officinalis Siebold & Zucc.* 12 g, *Lycium barbarum L*. 12 g, *Schisandra chinensis (Turcz.) Baill.* 9 g, *Epimedium sagittatum (Siebold & Zucc.) Maxim.* 9 g, *Fritillaria thunbergii Miq.* 9 g, *Paeonia lactiflora Pall*. 9 g, *Pheretima* 12 g, *Perilla frutescens (L.) Britton* 9 g, *Ardisia japonica (Thunb.) Blume* 15 g, and *Citrus × aurantium L* 9 g, which were also reported in our previous studies [11,30]. Similarly, the components of BJF also included 12 Chinese medicines: *Astragalus mongholicus Bunge* 15 g, *Polygonatum sibiricum Redouté* 15 g, *Codonopsis pilosula (Franch.) Nannf.* 15 g, *Atractylodes macrocephala Koidz.* 12 g, *Poria cocos (Schw.) wolf 12 g*, *Fritillaria thunbergii Miq.* 9 g, *Pheretima* 12 g, *Magnolia officinalis Rehder & E.H.Wilson* 9 g, *Citrus × aurantium L.* 9 g, *Aster tataricus L.f.* 9 g, *Ardisia japonica (Thunb.) Blume* 15 g, and *Epimedium sagittatum (Siebold & Zucc.) Maxim* 6 g, as also shown in one of our previous studies [10]. YZF is composed of 13 Chinese medicines, including *Panax ginseng C.A.Mey.* 9 g, *Polygonatum sibiricum Redouté* 15 g, *Ophiopogon japonicus (Thunb.) Ker Gawl.* 15 g, *Schisandra chinensis (Turcz.) Baill.* 9 g, *Lycium barbarum L.* 12 g, *Rehmannia glutinosa (Gaertn.) DC.* 15 g, *Neolitsea cassia (L.) Kosterm.* 3 g, *Fritillaria thunbergii Miq.* 9 g, *Pheretima* 12 g, P*aeonia × suffruticosa Andrews 12 g*, *Perilla frutescens (L.) Britton* 9 g, *Stemona tuberosa Lour.* 9 g, and *Citrus × aurantium L* 9 g [12]. Plant names were verified according to the Kew search tool. However, due to *Pheretima* and *Poria cocos (Schw.) wolf* not belonging to the scope of botanical medicinal materials, they were verified by searching literature and “Chinese Pharmacopoeia”. APL was obtained from Shandong Xinhua Pharmaceutical Co., Ltd. (Shandong, China). *K. pneumoniae* (strain ID: 46114) was purchased from the National Centre for Medical Culture Collection (CMCC, Beijing, China). The herbs were identified and prepared in fluid extract [10–12]. This study was approved by the Experimental Animal Care and Ethics Committee of the First Affiliated Hospital, Henan University of Chinese Medicine (2012HLD-0001).

For the six replicate samples from each group, RNA was extracted and purified from lung tissues using TRIzol reagent and Qiagen RNeasy Micro Kit, and then was measured by Agilent Whole Rat Genome Oligo Microarray. Raw data obtained in the above process were preprocessed with Agilent GeneSpring GX software (version 11.0). Differential expression analysis between two of these groups was performed using Student’s *t*-tests. In this present study, a cancer risk assessment signature for patients with COPD was firstly identified, and its reliability was verified in independent data. Among the genes constituting the cancer risk signature, the previously produced gene expression data of rat were applied to identify COPD key genes and further identify the genes that were reversed after drug treatment. The resulting information could potentially provide some guidance regarding the treatment of COPD and the prevention of cancer.

### 2.3. Identification of the qualitative transcriptional signature

Between the gene expression data of lung cancer and normal lung tissues from the training set (as shown in Table 1), highly stable gene pairs with opposite REOs were identified as the signature to predict the cancer incidence-risk in patients with COPD (with a threshold of 90%).

For the genes detected in a specific type of tissue sample from training datasets, all genes were pairwise compared to select stable gene pairs. For two genes, such as gene A and gene B, in one sample, their REO pattern was identified as A > B (or A < B) if the measurement of gene A was larger (or smaller) compared with that of gene B. In this study, a gene pair was considered highly stable when the gene pair (A, B) had an identical REO pattern in at least 90% of samples. Among the samples from two groups, if one gene pair was stable in both of groups but with reversal REO pattern, this gene pair was considered a reversal gene pair. Finally, from all gene pair combinations, the reversal gene pairs were selected and this was considered the signature for predicting the cancer incidence-risk.

For the gene pairs contained in the identified signature, the REO pattern of gene pairs representing lung cancer was used to calculate the cancer risk score in patients with COPD. For each patient, the cancer incidence-risk score was defined as the percentage of gene pairs characterizing lung cancer among the gene pairs of the signature. Supposing the number of gene pairs of the signature was *m*, among which *n* gene pairs had REO patterns characterizing lung cancer in this particular sample, then the incidence-risk score is given by *n*/*m*. The property of the identified signature was then verified in samples of non-cancer patients with COPD at different disease courses from several datasets.

### 2.4. KEGG pathway enrichment

A total of 330 KEGG pathways consisting of 7838 genes were obtained from Kyoto Encyclopaedia of Genes and Genomes (KEGG) database [31]. The significance of the pathways was determined by hypergeometric distribution model, calculated as the following:

$$p=1-\sum_{i=0}^{k-1}\frac{\left(\begin{array}{c} m\\ i \end{array}\right)\left(\begin{array}{c} N-m\\ n-i \end{array}\right)}{\left(\begin{array}{c} N\\ n \end{array}\right)}$$

where *m* indicates the number of genes annotated in one given pathway, *n* indicates the number of interested genes, *N* indicates the number of genes detected by the high-throughput platform, and *k* indicates the number of interested genes in the given pathway.

## 3. Results

### 3.1. Identification of a molecular signature to build the cancer incidence-risk score

**Figure 1.**
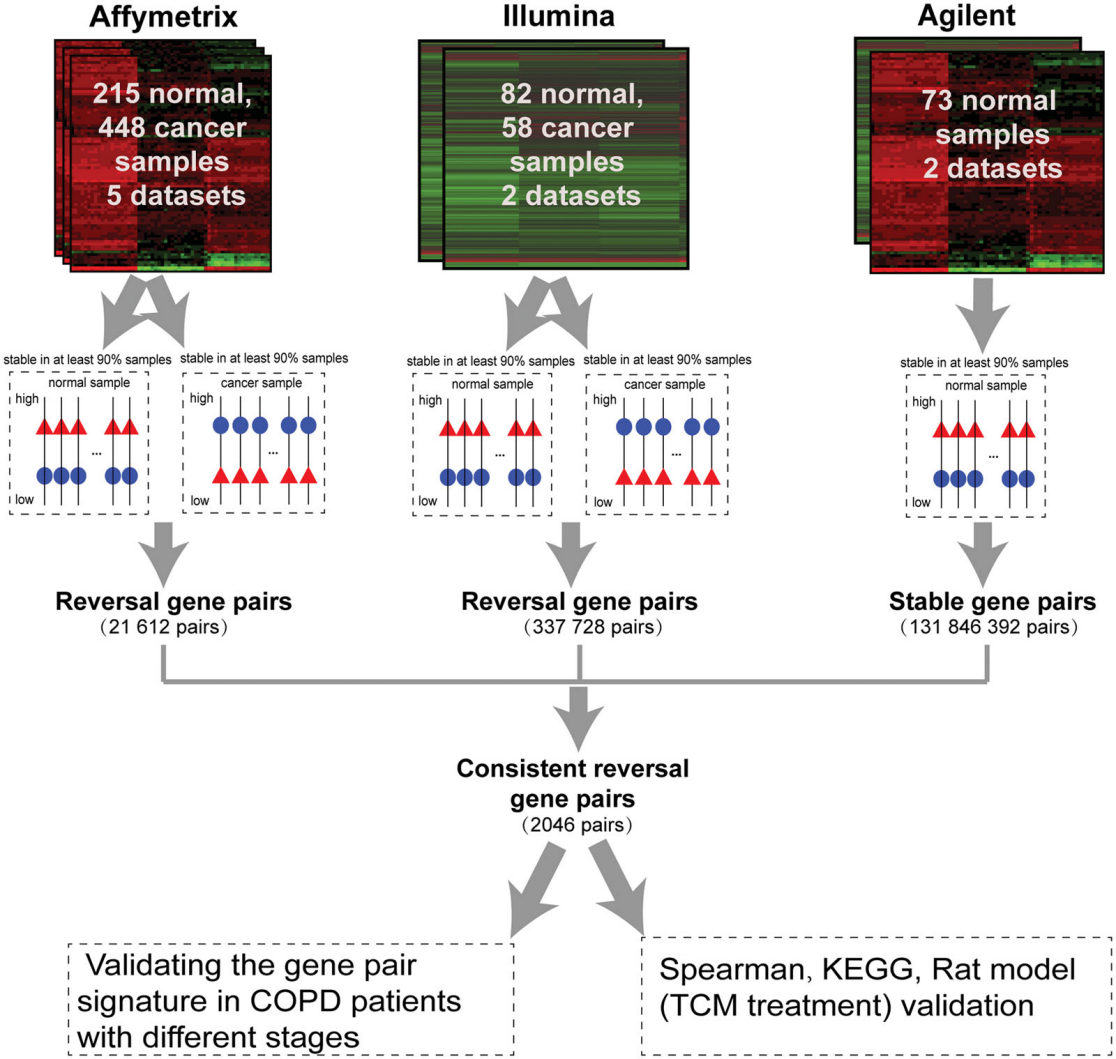
Analysis flowchart for this study.

The flowchart of this study is shown in Figure 1. Considering that lung cancer develops in a continuous, multistep process from normal lung tissues, with the threshold of 90%, stable gene pairs with opposite REOs between lung cancer and normal lung tissue samples were identified (see Materials and Methods).

For the 448 lung cancer and 215 normal lung tissue samples obtained from the five datasets detected by Affymetrix platform (as shown in Table 1), with the threshold of 90%, 21,612 stable gene pairs in both lung cancer and normal lung tissues but with reversed REO patterns were obtained; these gene pairs were considered stable reversal gene pairs. For the 58 lung cancer and 82 normal lung tissue samples obtained from the two datasets detected by Illumina platform (as shown in Table 1), 337,728 stable reversal gene pairs were obtained with the same threshold (90%). Among the two lists of stable reversal gene pairs obtained above, 3716 gene pairs were consistently identified. Based on those 3716 gene pairs, there were 2046 gene pairs with same REO patterns in more than 90% of the 73 normal lung tissues data detected by the Agilent platform. These 2046 gene pairs (Supplementary Table S3), including 1700 unique genes (Supplementary Table S4), were identified as the molecular signature and the percentage of the gene pairs characterizing lung cancer tissues were applied to predict the cancer incidence-risk score of non-cancer patients with COPD (see Materials and Methods). For a total of 506 lung cancer and 370 normal lung tissue samples in the training data, based on our signature consisting of 2046 gene pairs, the area under the receiver operating characteristic curve (AUC) value was 0.9929 (95% CI, 0.9634–1) (Supplementary Figure S1). The performance of the signature was then evaluated among patients with COPD from multiple datasets by comparing cancer risk scores of patients with COPD at different disease courses.

**Figure 2.**
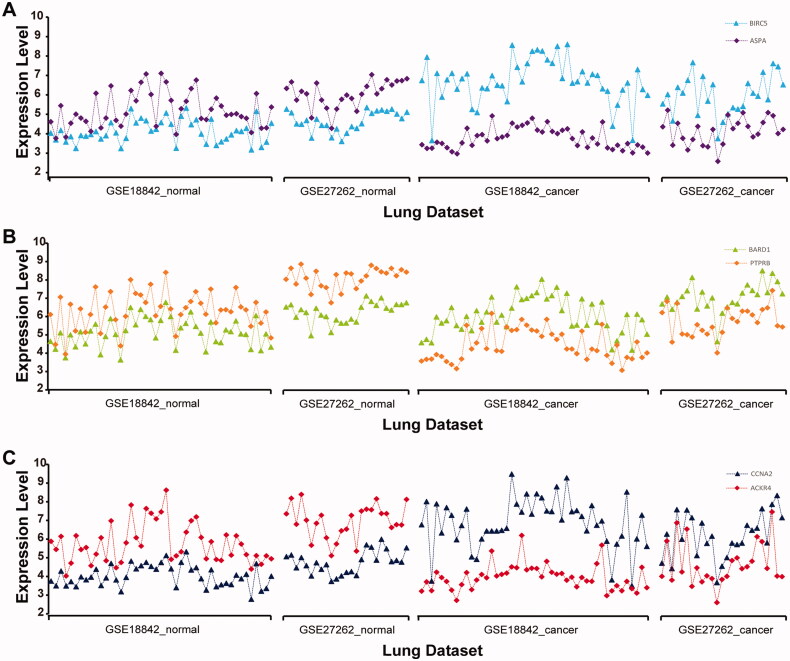
Distribution of gene expression levels for the three gene pairs—*BIRC5*-*ASPA* (A), *BARD1*-*PTPRB* (B), and *CCNA2*-*ACKR4* (C)—in GSE18842 and GSE27262 datasets. Horizontal coordinates represent cancer and normal lung tissue from datasets GSE18842 and GSE27262. Vertical coordinates represent the expression level of the corresponding gene.

Additionally, measurements of the genes ***BIRC5*** and ***ASPA***, ***BARD1*** and ***PTPRB***, ***CCNA2*** and ***ACKR4*** in lung cancer and normal lung tissue samples from datasets GSE18842 and GSE27262 were taken as an example to show that the qualitative transcriptional characteristics are robust in normal tissue samples (the expression value of ***ASPA*** (***PTPRB, ACKR4***) is higher compared with that of ***BIRC5*** (***BARD1, CCNA2***)) but reversed in cancer tissues (Figure 2). This would provide a basis for the selection of the cancer risk signature for non-cancer patients with COPD.

### 3.2. Performance of the signature in COPD samples at different disease courses

The pathophysiological process of lung cancer involves transforming normal lung to lungs affected by COPD, culminating in outright malignant transformation [9]. The performance of the signature (whose score ranges from 0 to 1) was therefore evaluated in COPD samples with different disease courses. Higher risk scores correlated with a greater cancer risk.

**Figure 3.**
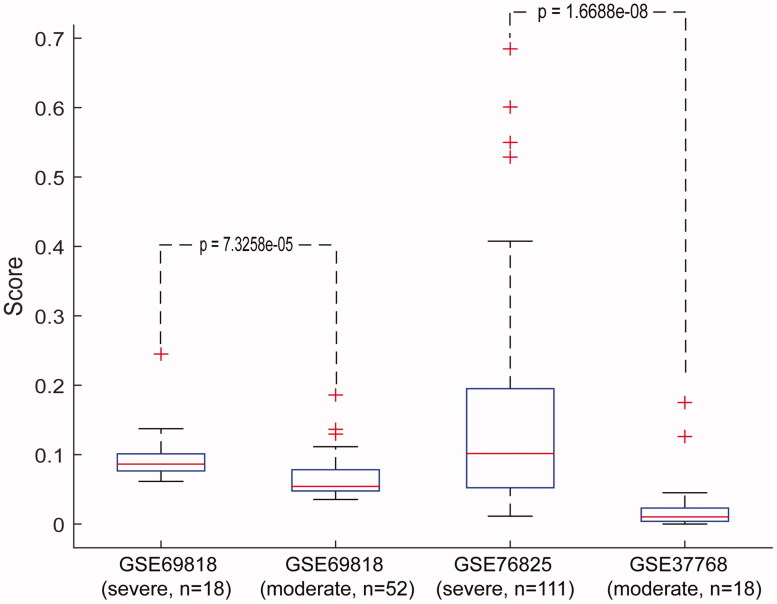
Performance of the signature in COPD samples with different disease courses. Horizontal coordinates represent severe and moderate COPD samples from public database. Vertical coordinates represent the score of our signature in severe and moderate COPD samples. The Wilcoxon rank-sum test was applied to calculate the *p* values.

In the dataset GSE69818, including 18 severe and 52 moderate COPD samples, the median of cancer incidence-risk score in severe COPD data was 0.0864, significantly higher than that in the moderate COPD samples (Wilcoxon rank-sum test; *p* = 7.33 × 10^−5^). In the datasets GSE76925, containing 111 severe COPD samples, and GSE37768, comprising 18 moderate COPD samples, similar results (Wilcoxon rank-sum test; *p* = 1.67 × 10^−8^) were obtained (Figure 3 and Supplementary Table S5). Moreover, the risk scores in samples from 18 patients with severe COPD that came from dataset GSE69818 were also significantly higher compared with those of the 18 moderate COPD samples from dataset GSE37768 (Wilcoxon rank-sum test; *p* = 2.39 × 10^−5^). Similar results were obtained in the analysis of severe and moderate COPD samples from datasets GSE76925 and GSE69818 (Wilcoxon rank-sum test; *p* = 5.40 × 10^−5^). These data suggest that our signature could be applied to various samples from multiple sources, highlighting the cross-platform performance of the signature.

### 3.3. KEGG pathway enrichment analysis based on signature genes

For the 2046 gene pairs in the signature, COPD stage-related gene pairs were further identified based on Spearman’s rank correlation tests. For the dataset GSE69818, consisting of 53 moderate and 18 severe COPD samples, 33 stage-related gene pairs were identified with false discovery rate (FDR) < 5%. Similarly, for the combined data of GSE76925 and GSE37768, 301 stage-related gene pairs were identified. For the above two lists of gene pairs, eight gene pairs were commonly identified and this was statistically significant (hypergeometric distribution model, *p* = 9.97 × 10^−2^). The two lists of gene pairs were then combined as the COPD stage-related gene pairs (a total of 326 gene pairs) for subsequent analysis, and 368 unique genes were included among those gene pairs. Based on these 368 genes and the hypergeometric distribution model, KEGG pathway analysis was performed. With FDR < 5%, no significantly correlated pathway was enriched, which might be ascribed to insufficient statistical power due to too few genes of interest. Therefore, pathway enrichment analysis was also performed under a relatively loose threshold condition. With *p* < 5%, six significantly related pathways were enriched (Supplementary Table S6) and these pathways are related to the progress of COPD. For instance, studies showed that there was 2.5-fold in COPD samples compared with the normal control samples for RNA polymerase II occupancy at the promoter [32]. CoQ10 or ubiquinone levels were decreased in patients with COPD, probably due to the defense response of the organism [33,34]. Beta-adrenoceptor-mediated lipolysis and thermogenesis are impaired in patients with COPD [35].

### 3.4. COPD-related genes in the rat model

The 368 human COPD stage-related genes identified above were ortholog converted to rat genes using the biological DataBase network [36], and 340 genes were obtained. Of these, only the measurements of 16 genes were significantly altered in the COPD rat model compared with control groups (six vs six) (Table 2).

**Table 2.** Differentially expressed genes between COPD rat model and control group.

Gene symbol	FC	*T*	*p*
*Dhx16*	0.530623	−3.72567	.003938
*Upf2*	1.039416	3.475603	.005965
*Uqcrc2*	1.015124	3.212685	.00929
*Rhobtb3*	0.884189	−3.09422	.011362
*Znhit3*	1.012759	3.046952	.012316
*Denr*	1.013586	3.017057	.01296
*Notch3*	0.975045	−2.97443	.01394
*Sdhc*	0.984546	−2.5892	.026987
*Dyrk2*	0.923138	−2.53068	.029836
*Sec61a1*	1.039209	2.52876	.029934
*Hmmr*	0.688459	−2.48504	.032263
*Exosc7*	0.943282	−2.48184	.032441
*Sh3gl3*	0.718159	−2.42208	.035933
*Noa1*	0.971469	−2.28776	.045186
*Trrap*	1.043465	2.252802	.047951
*Plk4*	0.857672	−2.24158	.048873

FC: fold-change of the COPD rat samples compared with control samples; *T*: test statistic value between COPD rat and control samples using the Student’s *t*-tests.

Among these 16 genes, those genes that were reversed when undergoing drug treatment (with BYF, BJF, YZF, and APL) were subsequently identified (Supplementary Tables S7–S10). The frequency of genes that were reversed after using this treatment protocol was then calculated (Table 3). With a relatively loose threshold (*p* < .2), the genes *Dhx16*, *Upf2*, *Denr*, *Notch3*, *Dyrk2*, *Sec61a1*, *Hmmr*, and *Noa1* were reversed in at least three treatment protocols and most of these genes were reported to be related to COPD or lung cancer [37–41]. The value of these genes warrants further study in the future.

**Table 3.** Frequency of genes that were reversed between the treatment and model group.

Gene symbol	FC (M_vs_C)	*T* (M_vs_C)	*p*_value (M_vs_C)	*p*_value (BYF_vs_M)	*p*_value (BJF_vs_M)	*p*_value (YZF_vs_M)	*p*_value (APL_vs_M)	Num *p* < .05	Num *p* < .1	Num *p* < .2
*Dhx16*	0.530623	−3.72567	.003938	0.701224	5.52E-09	0.097164	5.50E-05	2	3	3
*Upf2*	1.039416	3.475603	.005965	.027316	.011337	.058669	.02064	3	4	4
*Uqcrc2*	1.015124	3.212685	.00929	.022126	.043653	.93704	.822763	2	2	2
*Rhobtb3*	0.884189	−3.09422	.011362	.78651	.197268	.106184	.211585	0	0	2
*Znhit3*	1.012759	3.046952	.012316	.560379	.689214	.271842	.713968	0	0	0
*Denr*	1.013586	3.017057	.01296	.010532	.020327	.118537	.100091	2	2	4
*Notch3*	0.975045	−2.97443	.01394	.475693	.000212	.088364	.020111	2	3	3
*Sdhc*	0.984546	−2.5892	.026987	.728516	.001616	.317634	.000346	2	2	2
*Dyrk2*	0.923138	−2.53068	.029836	.718672	.000155	.19656	.000206	2	2	3
*Sec61a1*	1.039209	2.52876	.029934	.202428	.056216	.006779	.014547	2	3	3
*Hmmr*	0.688459	−2.48504	.032263	.994814	.124428	.03643	.016983	2	2	3
*Exosc7*	0.943282	−2.48184	.032441	.492618	.000315	.509816	.000261	2	2	2
*Sh3gl3*	0.718159	−2.42208	.035933	.36409	.814336	.458153	.089266	0	1	1
*Noa1*	0.971469	−2.28776	.045186	.138333	.00598	.351014	.173025	1	1	3
*Trrap*	1.043465	2.252802	.047951	.656511	.036755	.571989	.012966	2	2	2
*Plk4*	0.857672	−2.24158	.048873	.547285	.018171	.724516	.011406	2	2	2

FC: fold-change of COPD rat samples compared with control samples; *T*: test statistic value between COPD rat and control samples using the Student’s *t*-tests; *p*_value: *p* value between the corresponding two group samples (including Model vs Control, BYF vs Model, BJF vs Model, YZF vs Model, and APL vs Model) using Student’s *t*-tests; Num: number of the corresponding genes occurring in the four treatment protocols with one certain threshold.

## 4. Discussion

Using qualitative transcriptional features, a signature for the cancer incidence-risk assessment of non-cancer patients with COPD was identified. The signature was subsequently validated in patients with COPD at different disease courses from multiple data sources. This method was successfully applied in a previous study for assessing colorectal cancer incidence-risk among patients with precancerous colorectal lesions [42]. Carcinogenesis of lung cancer is a continuous, multistep malignant transformation process from normal lung tissues. One of pathogenic types of lung cancer arises from normal lung tissues advancing to moderate and then severe COPD, and eventually developing into cancer. The signature in the current study was developed based on normal lung and lung cancer tissue samples. Thus, the genes constituting the cancer risk signature might play vital roles in lung cancer or COPD pathogenesis. Based on the signature genes, key genes of COPD were further identified by correlation analysis and further optimized in control rat data and COPD rat model data with and without TCM treatment, which might guide efforts for cancer prevention and the treatment of COPD by TCM.

Most of the genes reproduced in the rat model were reported to be related to lung cancer or/and COPD. For example, dysregulation of *Notch1* and *Notch3* has recently been reported to be correlated with the pathogenesis of COPD [37]. The *Notch3* downstream target *HEYL* is an important regulator of airway epithelial cell proliferation and differentiation. Reduced expression of *HEYL* correlates with the impaired differentiation capacity of COPD primary human bronchial epithelial cells and overexpression of *HEYL* in COPD cells promoted differentiation into club, goblet, and ciliated cells [43]. Moreover, Sun et al. [1] found *Notch3* was downregulated in patients with COPD and could be targeted by miR-206. *Notch3* was also reported to be related to lung cancer. In three NSCLC cell lines (H292, A549, and Calu-3), Shi et al. [44] proved that overexpression of *NOTCH3* was related to increased cell growth rate, migration, and invasiveness abilities, as well as decreased apoptosis rate. Furthermore, si-RNA transfection in these NSCLC cell lines reversed these cellular biological behaviours [44]. *Notch3* can promote colony formation and sphere formation of stem-like capacity in lung cancer cells, and high expression of *Notch3* was related to a poor outcome of patients with NSCLC [45]. The missense mutation rate of *UPF1* or *UPF2* was higher in lung cancer [46]. *UPF2* binds *UPF1*, one of its family proteins, with a high affinity [47]. Through interaction with *UPF1* to promote *ZFPM2* mRNA decay, ZFPM2-AS1 could promote lung adenocarcinoma (LUAD) cell growth, migration, and the epithelial-mesenchymal transition process, thus exerting oncogenic functions [48]. The single nucleotide polymorphism (SNP) of rs115420460 in *DHX16* was significantly different in lung cancer samples compared with controls from the TRICL Consortium, and was demonstrated to be associated with lung cancer risk. Moreover, the location of this SNP was within the previously identified lung-cancer-susceptible region Chr6p21.33 and in high linkage disequilibrium with previously reported lung cancer SNPs from genome-wide association studies [49]. *DYRK2* might play an essential role in NSCLC, and its expression may predict the chemotherapy response in patients with NSCLC [38,39]. The expression level of *DYRK2* was significantly increased in lung cancer tissues compared with normal tissues, which might indicate a potential role of *DYRK2* in lung cancer development and/or progression [50]. Moreover, *DYRK2* was also overexpressed among lung cancer (LUAD and LUSC) in TCGA data [51]. *HMMR* is involved in lung cancer progression and is significantly associated with outcome [40,41]. *HMMR* is an independent risk factor for LUAD, and its high expression was significantly correlated with poor clinicopathological features and adverse outcomes (progression and metastasis of LAUD), whose expression may affect tumorigenic progression by altering the tumour microenvironment and playing a pivotal role in immune response regulation [52,53]. *DENR* was reported to be a risk gene in lung cancer, and its high expression could inhibit the survival of patients with lung cancer [54]. Further research on these genes might provide some valuable guidance for cancer prevention and TCM treatment of COPD.

On the other hand, our cancer risk signature in patients with COPD was developed based on normal lung and lung cancer tissue samples. Thus, the signature had the potential to discriminate lung cancer from normal lung tissues, and this ability was subsequently verified using independent data. Based on the majority vote rule, for the 59 normal lung tissues and 594 lung cancer tissues obtained from TCGA, the signature identified in the current study has excellent discriminating ability, and the values of AUC, sensitivity, and specificity were 0.9981 (95% CI, 0.6420–1), 93.64%, and 100.00%, respectively (Supplementary Figure S2). Similarly, for the 30 normal lung tissues and 36 lung cancer tissues obtained from GSE7670, the values of AUC, sensitivity, and specificity were 0.9991 (95% CI, 0.7441–1), 94.44%, and 100%, respectively. For the 30 normal lung tissues and 80 lung cancer tissues obtained from GSE43458, the values of AUC, sensitivity, and specificity were 0.9835 (95% CI, 0.6603–1), 80.00%, and 96.67%, respectively, and for the 20 normal lung tissues and 80 lung cancer tissues obtained from GSE33532, the values were 1.000 (95% CI, 0.5839–1), 100.00%, and 100.00%, respectively. These results demonstrated that the signature has the ability to discriminate lung cancer from normal lung tissues. Moreover, the performance of the signature was also validated in COPD-only patients and COPD patients with lung cancer by searching the gene expression data of lung tissues from these two groups of patients. One dataset (GSE8581) with COPD lung tissues from COPD patients with lung cancer, and three datasets (GSE103174, GSE151052, and GSE106986) with COPD lung tissues from COPD-only patients were obtained. There was no dataset that simultaneously contained lung tissue samples from COPD-only patients and COPD patients with lung cancer. Thus, the performance of the signature to predict cancer incidence-risk of COPD patients was validated in samples from different datasets. The risk scores in 15 COPD samples from COPD patients with lung cancer from dataset GSE8581 were significantly higher compared with those in 37 samples from COPD-only patients from dataset GSE103174 (Wilcoxon rank-sum test; *p* = 1.10 × 10^−8^). Similarly, the risk scores in COPD samples from GSE8581 were also significantly higher compared with those in 77 samples from COPD-only patients from dataset GSE151052 (Wilcoxon rank-sum test; *p* = 5.34 × 10^−10^). However, the risk scores were not significantly different between the 15 COPD patients with lung cancer from dataset GSE8581 and 14 samples from COPD-only patients from dataset GSE106986 (Wilcoxon rank-sum test; *p* = .68), which might be ascribed to low statistical power due to small sample size. These results further demonstrated that the signature could effectively predict the cancer incidence-risk of patients with COPD and also exhibited cross-platform ability.

Due to the lack of corresponding clinical follow-up data, it is not possible to verify whether the individuals without cancer and with high lung cancer incidence-risk score, as identified by the signature, eventually develop into cancer. Future studies will involve collaboration with affiliated hospitals to better evaluate the robustness of the signature; patients would be followed to further appraise the robustness of the signature and to compare the cancer incidence-risk score, calculated by the signature, with the time from diagnosis to carcinogenesis. This will determine whether individuals at high risk of cancer (calculated based on the signature) eventually develop into cancer. The financial burden of high-throughput sequencing is markedly decreasing. Consequently, for the scarce precious tissue samples at the clinical practice, it will be possible to simultaneously measure a set of disease genes that could more fully reveal the value of clinical samples under controllable cost conditions. Such data could be reused in other studies for different application scenarios involving diagnosis, histological classification, prognoses evaluation of disease, etc., thereby enhancing the value of the clinical research.

In conclusion, the molecular signature identified in this study (based on qualitative transcriptional characteristics) circumvents problems related to batch effects [55,56], variations in tumour epithelial cells from different sampling sites [57], partial RNA degradation [58], and amplification bias of minimum specimens [59]. The signature is suitable for inaccurately sampled tissues and can be applied for individualized analysis, which is more in line with the clinical setting [22]. Moreover, the reversed genes identified in the COPD rat model and drug treatment group might play a key role in medical treatment of COPD, and this warrants further investigation.

## 5. Conclusions

COPD is a common disease with severe health consequences. It is also a high-risk factor for lung cancer. For the non-cancer patients with COPD, it would be significant if their cancer incidence-risk could be assessed. Considering the unique merits of qualitative transcriptional characteristics (also called the within samples REOs of genes), which are insensitive to batch effects and could be used for the analysis of individual patients, a qualitative signature was identified to predict the lung cancer incidence-risk for non-cancer patients with COPD. Key genes for COPD were further identified, optimized by correlation analysis with COPD stage, and filtered in COPD rat model data. The genes that occurred in reverse fashion when the COPD rat model was treated with some TCM were further identified. In summary, the qualitative transcriptional signature circumvented problems associated with batch effects and is suitable for the individualized diagnosis of single samples, making it feasible for application in clinical settings for the surveillance of non-cancer patients with COPD. The value of COPD key genes in both TCM treatment of COPD and cancer prevention should be further explored.

## Supplementary Material

Supplemental Material

Supplemental Material

## Data Availability

The data that support the findings of this study are available on request from the first or corresponding author.
