# Supplementary material for: Identification of cancer risk assessment signature in patients with chronic obstructive pulmonary disease and exploration of the potential key genes
Source: Ann Med. 2022 Aug 20;54(1):2309–20. doi: 10.1080/07853890.2022.2112070 (PMC9415445; doi:10.1080/07853890.2022.2112070)

Figure S1 Area under the receiver operating characteristic curve (AUC) of the lung cancer and normal lung samples in training dataset.


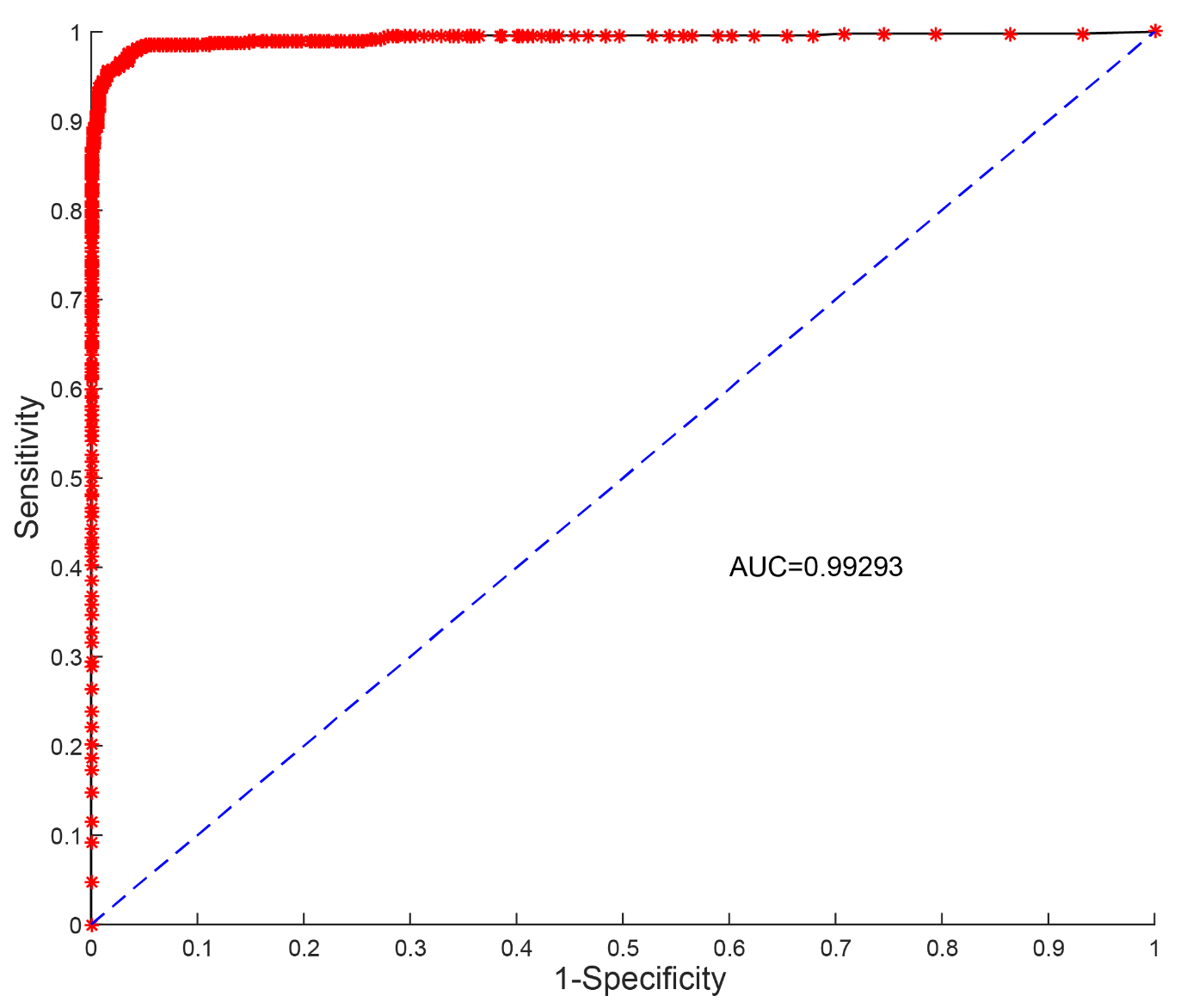


Figure S2 Area under the receiver operating characteristic curve (AUC) of the lung cancer and normal lung samples in the independent validation dataset from TCGA database (**A**), GSE7670 (**B**), GSE4348 (**C**), and GSE33532 (**D**).


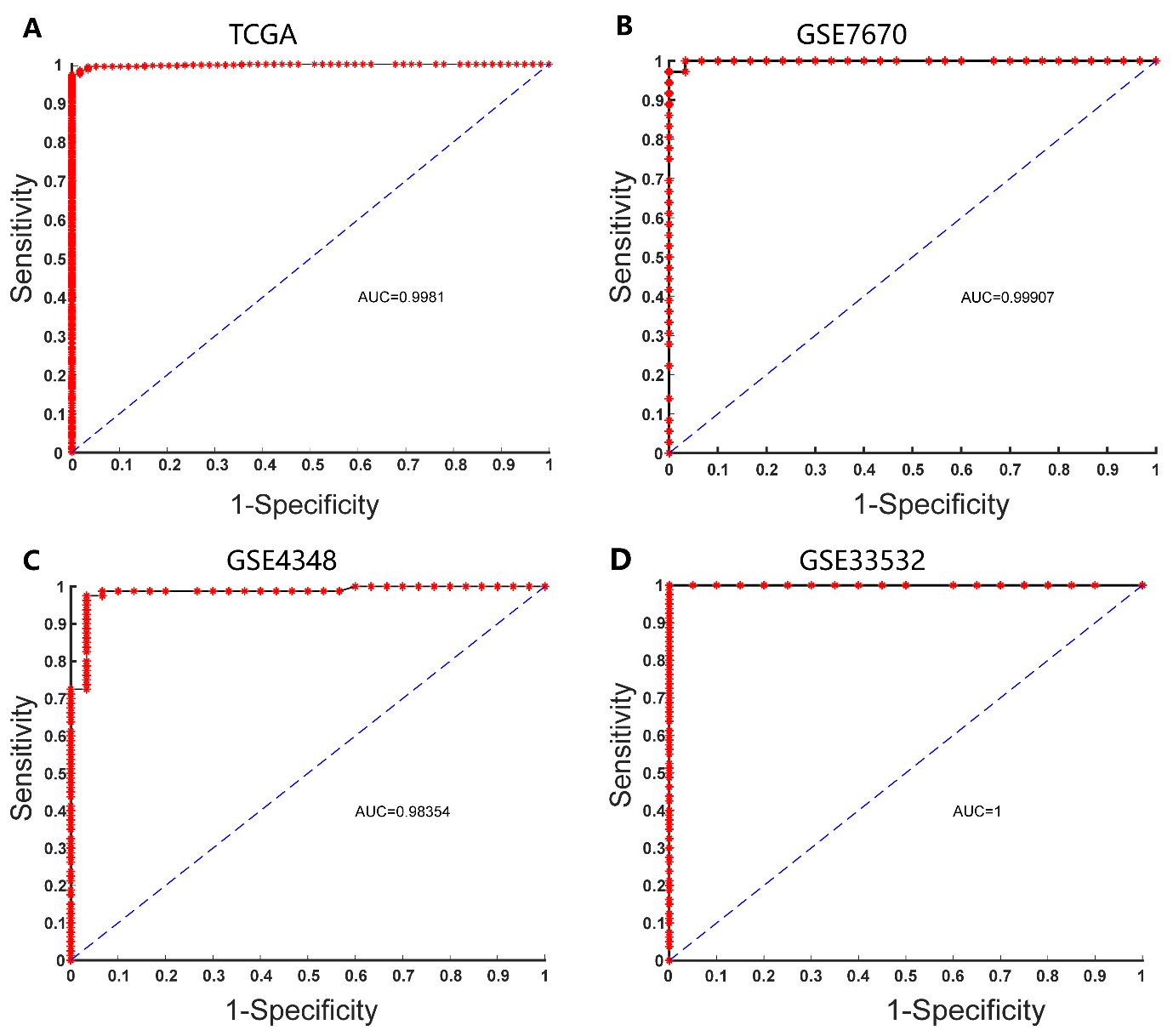

Supplement: Supplemental Material [file IANN_A_2112070_SM5275.docx]
